# Contextualizing mobility during the Ebola epidemic in Liberia

**DOI:** 10.1371/journal.pntd.0010370

**Published:** 2022-04-20

**Authors:** Mosoka Fallah, Stephen Lavalah, Tina Gbelia, Myers Zondo, Morris Kromah, Lucy Tantum, Gartee Nallo, Joseph Boakai, Kemoh Sheriff, Laura Skrip, S. Harris Ali

**Affiliations:** 1 Community-Based Initiative for Disease Surveillance and Sustainable Development, Monrovia, Liberia; 2 Youth Exploring Solutions in Liberia, Monrovia, Liberia; 3 York University, Toronto, Canada; National Institute of Infectious Disease, JAPAN

## Abstract

Based on findings from focus groups and key informant interviews conducted at five sites in Liberia between 2018 and 2019, we explore some of the key factors that influenced people’s motivation to travel during the 2014–2016 Ebola Virus Disease (EVD). We discuss how these factors led to certain mobility patterns and the implications these had for EVD response. The reasons for individual mobility during the epidemic were multiple and diverse. Some movements were related to relocation efforts as people attempted to extricate themselves from stigmatizing situations. Others were motivated by fear, convinced that other communities would be safer, particularly if extended family members resided there. Individuals also felt compelled to travel during the epidemic to meet other needs and obligations, such as attending burial rites. Some expressed concerns about obtaining food and earning a livelihood. Notably, these latter concerns served as an impetus to travel surreptitiously to evade quarantine directives aimed specifically at restricting mobility. Improvements in future infectious disease response could be made by incorporating contextually-based mobility factors, for example: the personalization of public health messaging through the recruitment of family members and trusted local leaders, to convey information that would help allay fear and combat stigmatization; activating existing traditional community surveillance systems in which entry into the community must first be approved by the community chief; and increased involvement of local leaders and community members in the provision of food and care to those quarantined so that the need to travel for these reasons is removed.

## Author summary

Human mobility is an important element of infectious disease outbreaks, both in terms of its spread and control. Understanding and dealing with the complex issue of human mobility warrants serious consideration in efforts to limit the spread of the disease. For these and other reasons, public health responses to infectious disease outbreaks must first be predicated upon a firm understanding of the contextual issues surrounding human movement and agency, especially those pertaining to the social, cultural and political-economic dimensions involved. Focus groups and key informant interviews conducted at five sites in Liberia in 2018/19 offered insight into the reasons people gave to account for their movements during the 2014/16 EVD outbreaks. Based on the understanding of mobility we gained from this study, we recommend that infectious disease response could be improved through the personalizing of public health messaging through the recruitment of family members and trusted local leaders to convey information that would help allay fear and combat stigmatization; activating existing traditional community surveillance systems in which entry into the community must first be approved by the community chief; and increased involvement of local leaders and community members in the provision of food and care to those quarantined so that the need to travel for these reasons is removed.

## Introduction

It has been noted that there are gaps in understanding how and why people moved during the 2014–2016 Ebola Virus Disease (EVD) epidemic in West Africa [1]. This lack of knowledge concerning mobility may have contributed to ineffective state and humanitarian interventions aimed at regulating population movement during the epidemic [1]. To address these deficiencies, this paper focuses on the social dynamics of mobility and how aspects of this dynamic contributed to some of the practical challenges faced in the EVD response. As part of this focus, we identify and discuss some of the factors that influenced mobility decisions and patterns during the 2014–2016 EVD epidemic in Liberia. The overall aim of this study is therefore to analyze the broader social, economic, and cultural elements that influenced mobility more generally during the EVD epidemic. In this light, we focus on contextual issues linked with two interrelated types of mobility. First, we examine the reasons people gave for their movement between communities. Second, we discuss the reasons people gave for evading mobility restrictions pertaining to the imposition of public health directives and quarantine measures. We separate these two types of factors for heuristic purposes in our analysis, but as we will see, they are in fact interrelated. We conclude with a general discussion of the implications our findings have for epidemic response.

The first officially documented EVD outbreak occurred in 1976 in a remote village in Zaire (now known as the Democratic Republic of Congo). Over the subsequent decades, numerous, smaller-scale EVD outbreaks emerged in similarly remote rural areas throughout Central and East Africa [2]. This geographic pattern of disease emergence in isolated and sparsely populated villages in this part of the continent, in part, limited the magnitude of the potential impacts of the outbreak [3]. In contrast, the 2014–2016 West African EVD epidemic was of an unprecedented scale. Notably, the epidemic took hold in the large and highly dense capital city regions of Guinea, Sierra Leone, and Liberia [4], and claimed more than 11,000 lives across West Africa [5]. The rapid spread of EVD in West Africa also demonstrated the speed at which the virus could travel across wide swaths of territory once it reached urban settings, particularly in settings where people had no previous experience with EVD with limited foundational preparedness and response apparatus in place [5]. Further, the epidemic took hold in regions where residents were known to frequently travel to other parts of the country by moped, bus, bicycle, boat on by foot, to tend farms, engage in trade or visit family members [5].

The index case of the West African EVD epidemic has been hypothesized to be a child who was infected by a bat in a small village in Guinea situated near the border with Sierra Leone and Liberia in June 2014 [5]. The village is located near major road networks, and it is thought that the virus was able to spread rapidly via trunk roads that connected the village to major cities of the two neighbouring countries, as well as the Guinean capital of Conakry [4]. From there, the virus spread to remote rural areas through capillary networks that were connected to the major urban centres [6]. These types of networks consisted of narrow, dirt roads that connected rural towns to semi-urban areas. The roads within these networks were typically made by a bulldozer or by hand on the basis of the collective efforts of rural residents. Although these roads were not commonly used by large vehicles, over the last few years there has been an increased rate of motorbike traffic on these routes [5, 7].

Though focusing on the larger issue of migration, Alexander et al. [8] have identified population movement as a major driver in the explosive spread of EVD in West Africa. To support this assertion they note that human movement (pertaining to both mobility and migration as interrelated phenomena) in West Africa has certain notable characteristics: (i) migration rates are seven times higher than the rest of the world; (ii) an estimated 11% of West Africans live outside their country of birth with between 30% to 40% residing outside the district or village of birth; (iii) there is a large displacement of people–for instance, in Liberia 54% of the over the age of 14 are identified as being internally displaced; and, (iv) in recent years there has been a significant increase in the proportion of people living in urban environments. To account for these trends in the midst of an outbreak is not a simple matter, particularly in post-conflict settings that lack resilient health systems and have limited infrastructure for surveillance and response activities. There is sometimes a tendency to attribute collective migration to emergent health crises alone, but this is misleading because such migration usually occurs within the context of a larger scale (temporally and often geographically) humanitarian crisis in which more immediate threats to life trigger such movement [9]. Others have pointed out that there is large scale migration that occurs between harvest periods in West Africa. During that time, young men in search of work use existing dirt/informal roads to avoid immigration checkpoints which makes it difficult to keep track of travellers and travel patterns. Researchers further contend that in West Africa the ECOWAS treaty allows migration without the need for visas [7]. Furthermore, Onoma notes that, “The widespread resort to flight as a means of escaping and resisting various pre-colonial, colonial and postcolonial state impositions across Africa has reinforced state apathy towards ‘unfettered’ mobility” [10]. In this light, mobility patterns are conditioned by broader historical forces at play that effectively, though perhaps inadvertently, encourage a culture of mobility. Such historical influence results in the development of particular types of cultural norms that inform everyday mobility choices and patterns. In sum, mobility decisions do not occur in a historical or cultural vacuum.

Despite their significance in how outbreaks may unfold, social and culturally-based understandings of the extent to which mobility contributes to disease spread has been lacking. Notably, it has been argued that existing analyses of mobility and Ebola control tends to treat mobility in reductionist terms, that is, mobility is treated as a phenomenon dis-embedded from the cultural context in which it is immersed [10]. As an analytical corrective it becomes necessary to learn more about the details of each social setting with reference to who is moving, how movement is planned, organized and implemented, where people are moving to and their reasons for moving. Without such a contextual understanding, people’s movement is not accounted for in experiential terms, but exclusively in structural terms, for instance, with reference to broader yet ill-defined concepts such as “displacement due to civil war” or “poverty”. Here, reference to civil war and poverty come to essentially function as proxies for much broader forces that require much greater and finely grained attention and interrogation that recognizes changes over time and space. In other words, paying attention to how human movement is influenced by a complex suite of social and economic factors.

## Methodology

### Ethics statement

The research protocol was approved by the Human Participants Review Sub-Committee of the Office of Research Ethics at York University (Toronto) and the National Research Ethics Board of Liberia (JFK Medical Center, Monrovia). Formal verbal or written consent was obtained from all participants.

The research presented here is based on a larger study that collected qualitative data concerning the factors that contributed to the relatively successful community-based response in Liberia during the 2014–2016 outbreaks. This type of response was based on the active participation of community leaders and members in various and wide-ranging aspects of outbreak response, including active surveillance, community messaging, and providing supplies and support for those quarantined [11]. These data on the community-based approach adopted in the West African context were collected to assist with the EVD outbreak response in the eastern Democratic Republic of the Congo in 2018–2020 through workshops held with the response teams in that country in July 2019. As such, our interview and focus groups guides were designed to collect data on Liberian and Sierra Leonean community members’ views of various aspects of the response and included questions such as: “Tell us about how Ebola affected your community, what changes did it cause during the outbreak and after the outbreak?” What aspects concerning the Ebola response made you feel bad and why?” What in your opinion was good about the Ebola response?” “In what ways did you resist or facilitate the response?”. To keep the present analysis manageable in scope, we focus here on the data we have collected from Liberia and we have selected out for analysis, those themes that dealt with mobility patterns within that nation, rather than with neighbouring countries, though we will on occasion make reference to cross-border mobility for the purposes of illustrating a particular point raised in the discussion. We do recognize that cross-border mobility is an important and complex phenomenon that presents unique challenges for disease response, and we hope to develop our work on cross-border mobility in a separate paper in the future by drawing on qualitative data from both Sierra Leone and Liberia as well as a separate qualitative dataset pertaining to the Democratic Republic of Congo and Rwanda. For preliminary work on this topic see [12].

**Fig 1:**
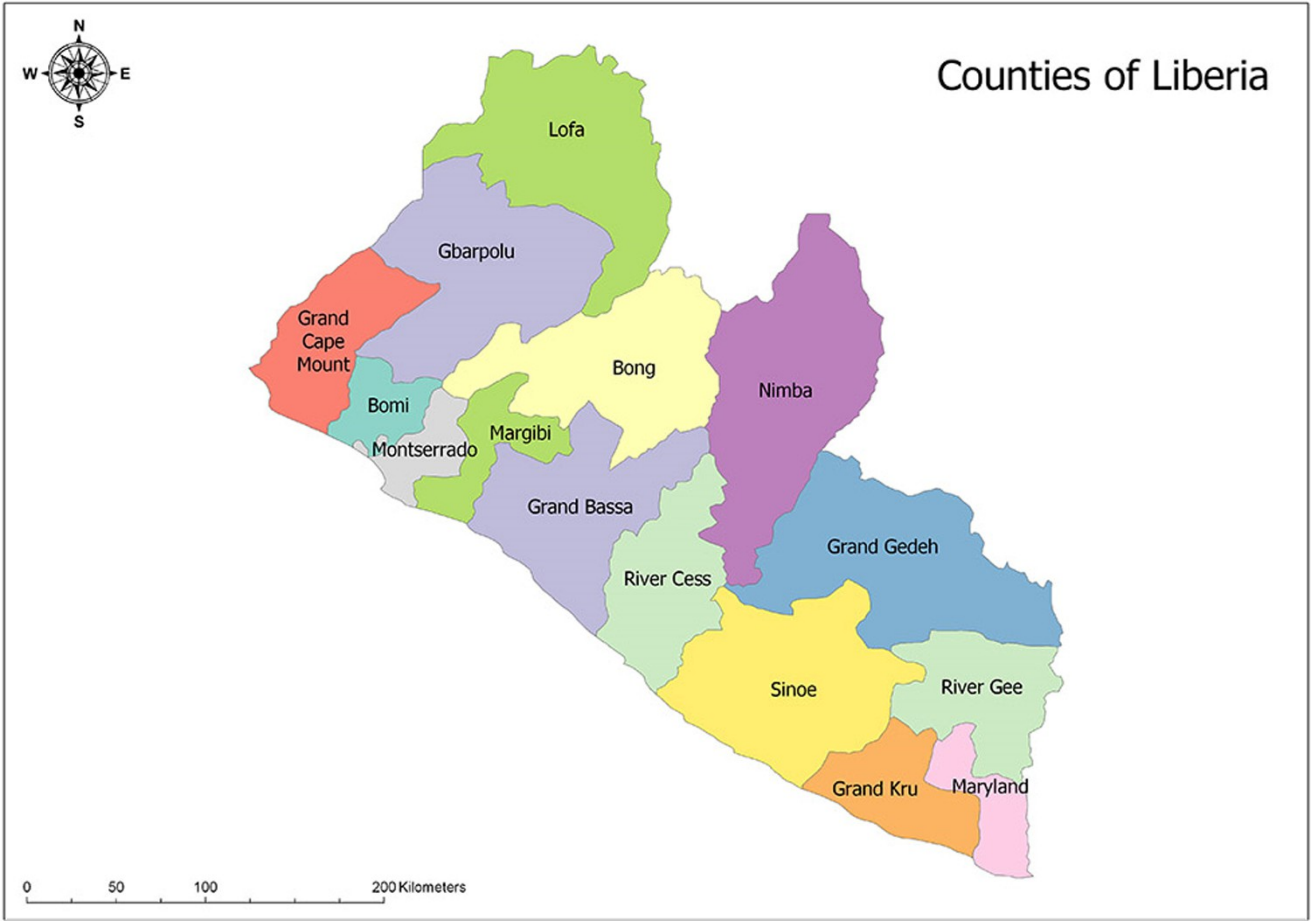
Counties of Liberia; Source: http://www.mapsopensource.com/liberia-counties-map.html.

The research was conducted in five communities within three Liberian counties—Montserrado, Lofa, and Margibi (Fig 1). The communities were selected from these particular counties because these three counties exhibited different levels of success in relation to adopting the community-based approach as an intervention in the response. Previous to the introduction of the community-based approach to response, the EVD response was based on a top-down strategy in which government and NGO-officials issued public health directives in a command-and-control manner–a situation that did not prove to be effective in keeping the case count down [13, 14]. All selected locales were known EVD “hotspots”. Specifically, based on earlier work, it was found that in Lofa County the community-based intervention proved to be initially unsuccessful and the disease spread from rural to urban areas, whereas in Margibi County the community-based intervention was successfully implemented during a period of resurgence [13]. In Montserrado County, mobility led to EVD spread from low- to high SES communities within the capital city of Monrovia [14].

The purpose of the present paper is not to do a comparative analysis of the three settings with respect to the mobility; that specific type of analysis will be pursued in the future. For the present analysis we cast a broader net to help understand the mobility dynamics during the EVD epidemic at a more general level. That is, our aim is to gain an understanding of how mobility unfolded during the epidemic and to investigate the reasons people gave to account for their mobility at a time when travel prohibitions were implemented as part of the epidemic response.

The communities of Banjor, Nyaford Town, and West Point are located in Montserrado County and are all within or near the vicinity of Monrovia; Needowein is located in Margibi County (situated southwest of Monrovia in the adjacent county) and Foya—a town in Lofa County, Liberia—is located about 450km from Monrovia in the northwest corner of Liberia near the junction point with Sierra Leone and Guinea.

The communities are multicultural with both Christian and Muslim religions. Inhabitants of Lofa include individuals from the Kissi, Lorma, Gbande, and Mandingo tribes, while people in Margibi are primarily from the Bassa and Kpelleh tribes. Inhabitants of Montserrado represent tribes from all 15 counties of Liberia. The livelihood of people in the five communities includes agriculture and business for residents of Foya and Nyanford Town, fishing activity and petty trade in West Point and Banjor, farming, charcoal burning, and fishing for residents of Needowein, and fishing and petty trade in Banjor. Residents of these communities routinely travel by commercial vehicles, tricycles and motorbike.

The researchers conducted a focus group involving 10 participants from each of the communities in this study. The focus group discussions lasted about 90 minutes. Interviews with 10 key informants were also conducted at each community site and these took 30 to 60 minutes to complete. Key informants included community leaders (e.g., local chiefs, religious leaders) and those involved in the EVD response such as active case finders and contact tracers. Both groups of participants were asked identical questions, but we were able to ask follow-up questions and request further elaboration in the case of key informants as a natural part of the semi-structured interview process. All focus group discussions and interviews took place in late December 2018, except for the West Point focus group, which was done in October 2019. As such, our data are based on retrospective accounts and reflections of community members’ experiences about three years after the EVD epidemic. For recruitment, community leaders, along with representatives of the NGO–Community-Based Initiative (CBI), were involved in selecting participants. The CBI group was formed during the EVD response by the first author (Fallah) to recruit, train and engage local leaders and members of the community in the EVD response, including involvement in such activities as: identifying possible cases and contacts, monitoring the status of cases and contacts, and support for those quarantined [11]. The participants actively recruited for the focus groups were: EVD survivors, individuals who had acted as caregivers of EVD cases during the outbreak, family members of deceased cases and response workers during the outbreak and community leaders who had been involved in the response. All participants were 18 years and above at the time of the research.

The researchers met with the community leaders to discuss the aims and objectives of the research and to ask for permission to conduct interviews with community members. Consent was obtained from all participants. The consent form was read aloud by one of the researchers to participants who could not read. Individuals voluntarily agreed to participate in the research after the aims and objectives of the research were discussed. The interviews were conducted in semi-private places such as in the front or backyards where people lived or in the offices of NGOs, and participants’ names were not recorded during the interviews to ensure confidentiality. For the focus groups, each respondent was provided a coded number to avoid disclosure of identity and ensure confidentiality. Participants were compensated for their time with US $5.00. Information was recorded through a portable voice recorder.

Focus group discussions were transcribed and translated from the vernacular to standard English. Liberian Kreyol English was used at all sites except Foya in Lofa County where an interpreter helped translate questions to Kissi for participants. The transcripts were systematically coded using the thematic analysis approach. The reasons for movement before, during and after the EVD outbreaks were identified and served as the basis of our subsequent analyses. Our orientation to coding and thematic analysis was initiated by a deductive approach where we first coded for various predetermined aspects related to movement, such as: types of movement (e.g., walking, motorbike, car, bus), reasons for movement and barriers, problems and challenges faced in movement. Once these broader groupings were identified we inductively analyzed these broader themes and discerned two specific subthemes. The first was “Site-to-Site Mobility”, which then served as thematic category for which we then coded the reasons for that type of mobility, such as familial and social obligations, stigmatization, and the search for what people perceived to be safer places. The second subtheme that was discerned pertained to mobility issues related to the imposition of quarantine during the epidemic—“Quarantine Related Mobility”. Under this subtheme we coded for those factors that were mentioned frequently or appeared to be emphasized by those interviewed–namely how the restrictions on mobility impacted the ability to earn a livelihood during the quarantine, and relatedly, how efforts were made to evade the mobility restrictions for various other reasons (as discussed in our analysis).

## Results

As mentioned, the thematic analysis revealed two types of mobility themes and we discuss each in turn.

### I. Site-to-Site Mobility

The reasons given for people’s movements during the epidemic varied but tended to be based on personal criteria, involving such interrelated considerations as: familial and social obligations, stigmatization and the search for a safe haven.

#### (a) Familial and social dimensions

An important criterion used in deciding where to relocate was *who* would be there at the chosen location to receive and allow the migrants to temporarily settle. Unsurprisingly, those sites selected for relocation were those places where relatives lived. However, during the epidemic, travellers would have to abide by local by-laws before being allowed by the community chairperson to enter the new location (a point to which we will return later in discussing quarantine measures). Thus, one resident from Needowein, whose brother was an EVD survivor noted:

So, I had to leave from where I was from to move to my auntie’s place in Soul Clinic [suburban area just east of Monrovia]. My auntie requested that my brother’s clearance from the Ebola Treatment Unit should be provided to her as a prerequisite for accepting us into her home because the community chairman requested such a certificate from strangers. I alongside my brother and children lived in Soul Clinic for one year until Ebola subsided.

Movement to other community sites took place to avoid contact with those members of the family who were ill. As Allen et al. [15] observe, this movement out from the family home may have resulted in the sick being left to fend for themselves. One community member from Needowein who became ill, but was unsure if the cause was Ebola, recalled that:

When I started to vomit, I told my wife that she and the children needed to leave the house. I wanted to be alone because I began to experience some unusual signs and symptoms similar to that of Ebola. My wife took our children away to [another community] until outbreak was over. Even though I was not severely sick, nobody came close to me.

Travel practices such as those described above do have profound implications for disease spread, thus it was noted by an EVD survivor from Foya County, Liberia, that:

A woman from our community who attended her son’s graduation in Monrovia came back with a strange sickness and that is the way we got the Ebola virus disease.

This quote reveals a noteworthy aspect of the disease spread. The woman referred to above resided in an area that was situated close to the border with Guinea and Sierra Leone. The epicentre of the West African EVD outbreak was thought to be in this vicinity–specifically in a rural village in Guinea located about 55 km northeast of Foya. Yet this patient acquired the disease from Monrovia and not from her own locality, despite the proximity of her place of residence to the epicentre. Thus, the chain of transmission was not what would have been expected, that is, her infection was not acquired through local transmission in Foya. Further, it is worth noting that the mobility route involved a round trip—starting from a rural region, visiting an urban centre, and then returning to the original rural region. Attention to mobility in Africa tends to focus on the unidirectional movement of people from the rural into the urban centre. This is especially the case in reference to displacement and movement of refugees during periods of crisis such as civil wars–and usually for the purposes of permanent settlement (and quite frequently this settlement occurs in informal settlement areas of large cities). Though relatively under-analyzed, movement from the urban to the rural does also occur, and not just in the West African EVD context. For example, during the 2003 SARS outbreak close to one million people left Beijing to return to their family villages with the intention of returning to the city after the crisis had subsided [16].

Past research has noted how West African burial practices, as part of one’s familial and social obligations, were particularly important risk factors in EVD transmission [5, 17, 18]. It is clear that the washing of the deceased body prior to Muslim burial and the embrace of the deceased by Christian and other religious groups facilitated the rapid spread of the disease. Funerals were super-spreader events because multiple people would be in attendance for the burial and in contact with the body of the deceased. This coincided with the stage that Ebola is most infectious: within the one to two day period before and after death. People would attend the funeral then return to their homes, sometimes in adjoining villages and further, thus contributing to another axis for mobility during the time of the epidemic.

Burial practices were a particularly sensitive issue during the early stages of the outbreaks because of numerous claims by community members that official EVD responders did not handle the deceased bodies respectfully nor did they allow the religious rituals to be carried out. Further, in the case of Monrovia, bodies were taken to be cremated which was not in accord with the religious norms of the families of the deceased [11]. This led some members of the community to hide the bodies of the deceased so that burial according to the traditional religious prescriptions could be carried out in a clandestine manner [11]. Thus, one member of the Banjor community noted that:

What made me feel really bad was that when a relative died, the response team came and carried the body away without telling us where the body was being buried. To this date, some people do not know where their relative is buried. So, some people started to hide the dead bodies and started doing secret burials.

Such practices had implications for mobility during the epidemic because the bodies of the deceased would need to be transported to certain parts of the settlement, often remote areas away from public scrutiny. The movement of the deceased in this way was a serious problem because it could facilitate continued EVD spread while simultaneously thwarting the case identification and contact tracing efforts needed to stop the outbreak. With public recognition of the urgent necessity of addressing this problem, a compromise strategy based on mutual understanding was forged between official responders and community members through the development of “safe and dignified” funeral arrangements in which family members garbed in personal protective equipment could witness the burial rites in person [6]. Further, the need to travel to attend burial ceremonies may have also lessened because of better communication between responders and community members. For instance, it was noted by a Lofa County resident that:

So, the white people asked us if we would agree to bury those who died here to be buried in our own town. That we would not carry them to Foya for burial. The chiefs called a meeting and agreed to this.

#### (b) Stigmatization

As explained in previous research on EVD in Western Africa [19], stigmatization is not a trivial matter but a social phenomenon with significant implications for epidemiological field investigations and outbreak response. This is because stigmatization may influence individuals to be reluctant about disclosing information about their infection status and/or possible exposure, thereby confounding contact tracing initiatives and allowing cases to remain undetected (see for instance: [20] with reference to tuberculosis and [21] with respect to SARS).

In the present case, it was found that individuals, families and survivors were stigmatized, as were community members who lived close to those infected. Furthermore, places or communities may themselves became stigmatized. The following excerpts illustrate some of the ways stigmatization was experienced during the outbreaks and how it served as the impetus to relocate.

One individual from Foya, Lofa County who had a family member with EVD noted that stigmatization rendered him a pariah, so much so that he was shunned at the marketplace and was not even able to purchase food. As a consequence, he felt compelled to move:

Even when we went to buy people rejected our money. They complained that the virus was on our hands and therefore on the money. We caught hard times and difficulties. I had to move to survive.

An individual from Needowein, Margibi County noted that sometimes entire families affected by Ebola would become stigmatized in such a way that their life in the community became unbearable from the shame and disgrace they received, thus forcing the entire family unit to relocate. Further, other community members who were not infected but lived within the proximity of Ebola-affected families also felt they had to move elsewhere because of concerns over discrimination:

Families were affected, and based on the stigmatization they received, the only way they could deal with that was to change their environment. Sometimes, even those who had some association with infected families felt they had to move. So that you could get away from the shame and disgrace.

Thus, stigmatization was sometimes the result of simply association with those infected. We see this in the case of one individual from Needowein, Margibi County who noted that:

I was one of those that left my community during the Ebola crisis because I lost my parents. I left the community because I was tired of the fact that when I passed people, they were talking about me and pointing at me.

The public shaming that came with stigmatization therefore served as a strong impetus for relocation.

It was also found that sometimes, those travelling during the epidemic were viewed with suspicion and stigmatized. For instance, a religious leader from Banjor remarked that:

If you saw someone traveling, you needed to be careful with that person because you didn’t know if the person has contracted Ebola. People would presume that because you are travelling it means you are sick.

This notion that mobility implies infection may affect mobility patterns in two ways. First, it may serve as a deterrent to movement as people may reconsider their need to move because of the possibility of potential stigmatization they may face during their travels. Second, it may result in travel that is done in a more stealth-like fashion, as we see in the examples we discuss later, this is especially pertinent in situations where individuals attempt to evade quarantine (see Section II (b) Evading Mobility Restrictions).

#### (c) Finding a safe haven

Some were motivated to move in order to find a safer location to reside, or to find a place that they felt would offer better care:

People left a particular community once there were confirmed cases of Ebola being reported in that community. If they moved to a community considered as safe haven and people began to be infected by Ebola, they would again relocate to another community. Everyone was in search of a safer place for their families. (Needowein Resident 1)

Yes, I will say that people from other communities wanted to have come over here because they realized that this place was a safe haven. Some people heard that there were no cases of Ebola being reported here because of our own adherence to the health regulations and protocols. People from other communities wanted to have come here to stay until the Ebola subsided and everything returned to normal. (Nyanford Town Resident 2)

I can surely say that our community was a safe zone because many people were eager to run here for safe haven. When you heard that Ebola did not reach our community and no deaths was recorded here, it was because of our strict obedience to the health regulations and protocols. (Nyanford Town Resident 3)

Thus, in addition to moving for reasons of being with family during difficult times, or to escape stigmatization, we see that people were sometimes also motivated to relocate because they perceived the risk of EVD infection to be lower in other communities.

### II. Quarantine related mobility

#### (a) Impacts of mobility restrictions on livelihood

Urban researchers Muggah and Florida [22] observe that because informal settlements were usually located on the periphery of urban centres, residents frequently needed to travel to places outside their settlement areas to pursue their daily work. As such, many residents of informal settlements would travel to work in other parts of the city, sometimes over long distances, by jamming together and sharing rides in vans and buses–including as we will discuss in the last section, to work in more affluent neighbourhoods. This mode of transportation was the only means of affordable transport for those with very low incomes and no savings. Muggah and Florida [22] comment that such modes of travel however are “perfect vectors” for disease. During the EVD epidemic, to halt disease spread, this form of travel was suspended, and quarantines were implemented. During the ensuing periods of self-isolation and quarantine, food and money were still required despite the mobility restrictions. In particular but relatedly, the impacts of such mobility restrictions were significant in terms of the loss of livelihood. One community leader in Foya noted for instance that:

We could no longer commute in and out of Foya to conduct our businesses like before. Even farmers who cultivated farms in other communities affected by Ebola were afraid to visit their farms… Many people lost their sources of livelihood and income as a result some people started selling their properties just to survive.

While another member of the Foya community corroborated such an observation and went further to note a directly specific economic impact of the travel restrictions imposed:

During the Ebola outbreak, we could not go on our farms. Consequently, the birds ate all the rice since no one was there to drive the birds away. Our livelihood was destroyed.

The inability to travel to earn livelihood and secure adequate food were especially problematic if quarantine measures were adopted without warning. This was the case of the West Point informal settlement, when residents awoke one morning to face barricades set up by police and military officers as part of an unannounced enforced quarantine [23]. Being deprived of essential items needed for survival led some to feel that they were put in a desperate position and “backed against a wall”. This resulted in intensified tension between community members and governments forces that eventually led to standoff between the two groups that led to the tragic shooting of a young boy by police forces at the site of the blockade. For instance, one focus group participant in West Point expressed the following view:

The government decided to come up with the harsh response of quarantining the area, blocking the area and with no food coming in, family members were not permitted to come in to visit their family to help, people were not allowed to go out to get food. So, these are the things that caused the riot.

As we will discuss further below, faced with these types of challenges related to food and livelihood, some actively sought ways to evade the imposed mobility restrictions.

#### (b) Evading mobility restrictions

In confronting the concerns and reasons discussed above, some individuals fled their places of residence in a clandestine manner to avoid detection by authorities. Thus, the extent to which quarantines were successful in limiting people’s movement varied in accordance with the particular circumstances found in different locales. For instance, even though travel blockades were established on roadways, this did not stop people from entering and leaving the district through the countless paths and roads that the state had no way of policing. Similarly, as Onishi [24] notes, it was possible for people in the West Point settlement to escape quarantine relatively easy through informal pathways, as one resident noted:

Since West Point is overpopulated and everyone lives in congested, misshaped houses, it is easier for someone to escape quarantine by passing through another person’s window and there could be absolutely no traces of the escapee. [24]

Thus, it was noted that during the quarantine period, one West Point resident “turned his living room into a tollbooth, charging others to escape through his apartment at the edge of the cordoned area” [24]. Meanwhile others were able to leave the peninsula on which the informal settlement was situated by simply swimming to the mainland each day, while some even bribed the soldiers enforcing the quarantine to allow them to leave (ibid). Interestingly, it is worth noting that people not only “broke out” of quarantined areas, but they also often “broke in” after their business outside the home was completed [10]. Thus, one member from West Point remarked that:

The street kids always wanted to leave West Point, but the soldier guys [that enforced quarantine blockade] would not let them go unless they gave a bribe. They would go out, do their hustle [i.e. street trade] and come back into the community by giving the soldier guy a bribe.

In other places, people evaded quarantine under the cover of the night, as described by a resident of Needowein, Margibi County:

Some people stated leaving overnight escaping quarantine and crossing the Gbein River (in Margibi County) in canoe; because they didn’t want to be in Dolo Town [a town in the same county under a military enforced government lockdown] and risk infection, so they decided to leave the area for fear of being stigmatized.

As alluded to in the previous section, one motivating factor for people to escape quarantine was to move to another location that they considered to be safer. Thus, one resident from Nyanford Town, Montserrado County noted that:

Yep, people wanted to come to my community because, as you can see, this community is not as populated compared to overpopulated West Point. So, people tried to secretly run away at night from West Point, New Kru Town and Logan Town to come here because they felt that this community is less populated.

## Discussion and conclusion

The reasons given for mobility during the EVD epidemic were multiple and diverse. Sometimes a conscious decision was made to relocate. The motivating factors behind the intention to relocate included: people’s fear of the threat of EVD infection and the notion that their current community was a place of higher risk compared to other communities that were perceived to be safer (because of fewer cases); the expressed need for individuals or families to extricate themselves from situations in which they were socially ostracized due to stigmatization; and the desire to be with members of the extended family during the unsettling experience of an epidemic. Movement, especially during the earlier stages of the epidemic, before the full magnitude of the threat was known, was driven by: the need to fulfill social obligations such as attending graduations and other rites of passage including burial rites; as well as the need to travel to obtain food and earn livelihood. It should be kept in mind that these factors were not discrete and mutually inclusive, rather they overlapped and reinforced each other in ways that influenced the various patterns of mobility that unfolded. For example, leaving a community out of fear of being infected meant that people were more likely to travel in a covert manner. This was because conspicuous travel during the epidemic meant a higher chance of detection and being reported to authorities for evading quarantine orders. The convergence of these types of mobility related reasons and issues highlights the complexity and challenges involved in developing contextually appropriate response measures in relation to the restriction of mobility during the epidemic. As such, Wilkinson [25] observes with reference to COVID-19 (but relevant to infectious disease spread more generally), that the swift adoption of strict mobility restrictions without proper consideration of the social and economic complexity involved, may inadvertently exacerbate disease spread. Thus, Wilkinson [25] notes how the rapid introduction of travel restrictions suddenly led to the populations fleeing or travelling under the radar, as was the case in northern Italy during the early stages of the COVID-19 pandemic. Similarly, Campbell [1] notes that insufficient attention was paid to the social and economic complexity of mobility during the EVD epidemic. Thus, although mobility was recognized as a critical issue by responders, this recognition resulted only in focusing narrowly on restricting movement through curfews, border controls and quarantine–rather than adopting an approach that could address the concerns that motivated people to move in the first place. Campbell [1] observes that these strategies based strictly on restricting movement resulted in confrontation, frustration and the resentment of authorities. To address such issues, it may be useful for intervention strategies to take into account the more personal or informal factors that undergird mobility. This would help ensure that localized considerations pertaining to mobility are better incorporated in epidemic response strategies. We proffer some suggestions in this light.

Addressing fear as a motivating factor in mobility could be addressed through careful public health messaging that targets the specific concerns of those affected as they go about their daily lives. To pursue such a strategy, several authors [26–28] have noted that public health messaging must take into account the fact that information from official government sources were sometimes viewed with skepticism and distrust because of local political and historical circumstances. Such perceptions in turn led to the spread of alternative explanations based on misinformation and conspiracy theories. To deal with issues of distrust and communication, it may be helpful to recruit well-reputed and locally known trusted leaders to convey valid information and messages to the community. For instance, leaders could help assure members of their community by communicating accurate information about the threat of EVD. In particular, such action may address concerns some community members had that their particular community was more at risk of EVD infection than other communities. Such an approach to messaging may diminish the fear-motivated inclination to relocate.

The recruitment of local leaders was found to help address the stigmatization that had motivated some to leave their communities. For example, one imam in the Banjor community revealed to us in an interview that he would make it a priority to go out and publicly embrace those survivors who returned to his village from the Ebola Treatment Units. This inspired confidence in bystanders by showing them that there were no grounds to stigmatize and shun those returning to the village. Such public displays in the community by the local leader perhaps also had the further effect of reinforcing in people’s mind the notion that EVD could be beaten, thereby providing some sense of optimism during grim times and underling the reality that having EVD was not a death sentence.

The imposition of travel restrictions was not necessarily found to be effective in curbing funeral attendance, and this was understandable if one situates the importance of this rite of passage in its proper cultural context [28]. During the later stages of the epidemic response, the importance of burial rites in West African culture was indeed recognized and measures were taken to reduce the infectious disease spread potential of funerals by ensuring public health protocols on hygiene, personal protection equipment, and physical distancing. This enabled burial rituals to be safely conducted in manner respectful to religious and cultural traditions while ensuring that people could travel to attend funerals in a safe way.

Although it may be difficult to halt people’s travel in relation to personally significant aspects of life such as attending funerals, other types of travel may be dissuaded by resorting to personal appeals. For instance, to discourage people from travelling to stay with family in other communities during the epidemic, Onoma [10] notes that those moving between places would often notify their relatives in advance of their trip. This form of communication could help discourage travel if the relatives replied to would-be travellers with messages such as, “Please don’t travel here if you’re sick, it could harm us.” This type of informal messaging Onoma [10] notes, is more effective when it takes on a more personalized tone from family and friends instead of receiving a formal message from the government that orders people to comply to a government directive not to travel.

The motivations behind decisions to move were, as we have seen, varied. But for some, relatives and extended family members were particularly sought out because it was with them that people understandably felt safe, or felt that they could receive care if they became ill. As such, people were willing to travel long distances to go to relatives. At the same time, many avoided travelling to Ebola Treatment Units to receive care [29]. As Farmer [30] notes, the decision to avoid Ebola Treatment Units was made because people realized that such facilities offered little in terms of providing care, and in fact, family members during the early stages of the response were not allowed to meet their ill relatives (later this was allowed through the use of safety precautions, such as physical distancing, personal protective equipment and transparent plastic barriers). Further, public health messaging during the earlier response phases did not seem to address the immediate concerns of those affected. For example, immediate concerns about receiving care were not addressed, rather, the messaging emphasized adopting strategies based on faddish concepts such as “cultural resilience” or being lectured on not eating bushmeat, even though most transmission occurred through person-to-person transmission. This type of misguided emphasis in public heath messaging occurred because, as Farmer [30] observes, the official response emphasis tended to focus on control and containment over treatment and care. Public health messaging and directives based on control and containment over care and treatment were simply not relatable or even relevant to many peoples’ situations. Under these conditions, it may be helpful to rethink the treatment aspects of the public health messaging as they relate to travel pertaining to the seeking of care. For instance, as alluded to above, family member, elders and trusted local leaders could be recruited to convey personalized messages about the different care options available, including related information about when to travel to seek care at Ebola Treatment Units, while at the same time emphasizing the importance of not travelling and respecting quarantine and isolation measures.

The issues emanating from evading quarantine measures, such as surreptitiously leaving and sometimes returning to the village or community, are more difficult to address and may never be wholly effective because of the myriad ways people may enter and exit such locations. However, one method that was adopted to deal with this issue involved the establishment of sort of a “neighborhood watch” system that could at least serve as some form of deterrence to evasive forms of mobility [17]. As Richards [17] notes, such a *de facto* surveillance system is based on the long-established and widespread West African institution of ‘landlord’ and ‘stranger’. This institutionalized surveillance system is based on the requirement that the presence of any stranger (i.e., visitor) had to be first reported to the village chief. The chief would then decide if the person was allowed to enter and reside in the community (even for a night). This was to ensure the proper treatment of the visitor as well the host. Under the EVD circumstance, this system was mobilized to collect information about all visitors, as the chairperson or chief of the village would take down the name and address of the visitor, as well as other pertinent health information such as whether the person was presenting symptoms and who they were in contact with. Such information could then be used to aid case investigation and contact tracing efforts as necessary, especially in helping to identify those who may be hiding [11]. Interestingly, Richards [17] notes that this long-established system of social scrutiny was enhanced in Sierra Leone (and by extension Liberia) because of the recent civil wars when villagers would adopt a vigilant stance to look out for possible infiltration by rebel spies. The onset of EVD, he observes, has further strengthened the legitimacy of this traditional form of monitoring. Our field research revealed a couple of instances where the community chairperson did not allow a visitor to enter the community to stay with relatives (thus forcing them to return to their home village). Although these instances revealed some level of tension and argument that arose with the confrontation between visitor and community chairperson, we do see that this type of community surveillance system has the potential to dissuade travel during the epidemic.

To address issues of mobility driven by the search for livelihood and food, during the later stages of the response, a community-based approach was adopted. In this community-based approach, community leaders were recruited to spearhead and organize the mobilization of community members in various aspects of the epidemic response, including providing food and water for those quarantined and support for those who were caring for quarantined family members, and conveying information to extended family members [for a detailed discussion of the range of these activities see [31, 32]. Such community-based response efforts helped quell the urgent motivation to travel outside the village or community because at least the basic needs for survival would be ensured.

Since the time of this writing, the context in which individual mobility unfolds during an epidemic has changed from the time of the West African outbreak discussed here. For instance, as noted by a reviewer of this manuscript, one significant development is of course the current availability of an Ebola vaccine. The introduction of the vaccine has led to different types of mobility issues that still nevertheless have implications for the types of issues we have raised in this paper, including those pertaining to public health messaging, addressing fear and the need for mobility restrictions. We cannot engage in a full discussion here, but one important question that has arisen in light of the new circumstances is the issue of vaccine hesitancy that was evident during vaccination campaigns pursued in the eastern side of the Democratic Republic of Congo in 2019 [33]. The relationship between vaccine hesitancy and mobility may be a salient and worthy of consideration as hesitancy may prompt people to flee their locations or hide to evade vaccination teams–an especially challenging barrier if rink vaccination strategies are to be pursued. Future research may reveal if this turns out to be a mobility-related issue of concern for Ebola outbreaks, as well as for the current COVID-19 pandemic.

The importance of considering factors pertaining to contextual mobility is also important to consider in the current COVID-19 pandemic. For instance, in preliminary research we have found that in Monrovia, the spread of COVID-19 and Ebola unfolded in opposite directions along the Socio-Economic Status (SES) gradient. Specifically, the spread of Ebola from low to high SES areas. People in low-SES informal settlements were the among those first infected. Significantly, some of those first infected by EVD were those residing in informal settlements. These included many who worked as drivers, maids, security personnel and cleaners for homes in middle-class neighbourhoods, where they would commute each day for their work. In this way, the disease spread from informal settlements to those living in the more affluent areas. In contrast, those first infected by COVID-19 consisted of the well-educated and wealthy. That is, those in a higher-SES strata who were in a position to travel internationally for conferences, meetings, and business. Upon becoming ill on arrival home, the disease was then transmitted to their employees–that is, again, the drivers, maid, security personnel and cleaners, who this time would often act as caregivers. Once infected these workers would bring COVID-19 back with them to the informal settlement.

Finally, we note that our study has at least two limitations. One limitation is that questions about mobility were not specifically asked. We believe however that the data collected nevertheless allowed for valid inferences about mobility. Comments made about mobility came out naturally in an unsolicited manner as people discussed their experiences with different aspects of the EVD response as part of the investigations of the larger study on which the present more delimited study was based. The larger study for instance focused on such issues as: stigmatization, trust in response officials, difficulties faced, and so on, in which aspects of mobility issue were expressed incidentally. Lastly, it may be noted that during the time of the epidemic itself or shortly thereafter, people may have been reluctant to speak about their specific mobility patterns because of fear of receiving sanctions for violations of outbreak response directives. However, fears related specifically to mobility would likely have lessened over time as the interviews and focus groups were conducted several years subsequent, thus minimizing the threat of sanctions because such threats were no longer applicable in the current day. A second limitation pertains to the purposive sampling pursued in this study. Such sampling is not geared to generalizability to a population, but rather is focused on gaining insights to advance theoretical and conceptual frameworks. As such, the main objective of the present study was to gain qualitative and theory-informed insights into characterizing patterns of mobility and reasons for mobility during the EVD epidemic rather than to generalize to all epidemic situations *per se*.
